# Supplementary figures and images for: Constraints on microbial communities, decomposition and methane production in deep peat deposits
Source: PLoS One. 2020 Feb 6;15(2):e0223744. doi: 10.1371/journal.pone.0223744 (PMC7004313; doi:10.1371/journal.pone.0223744)

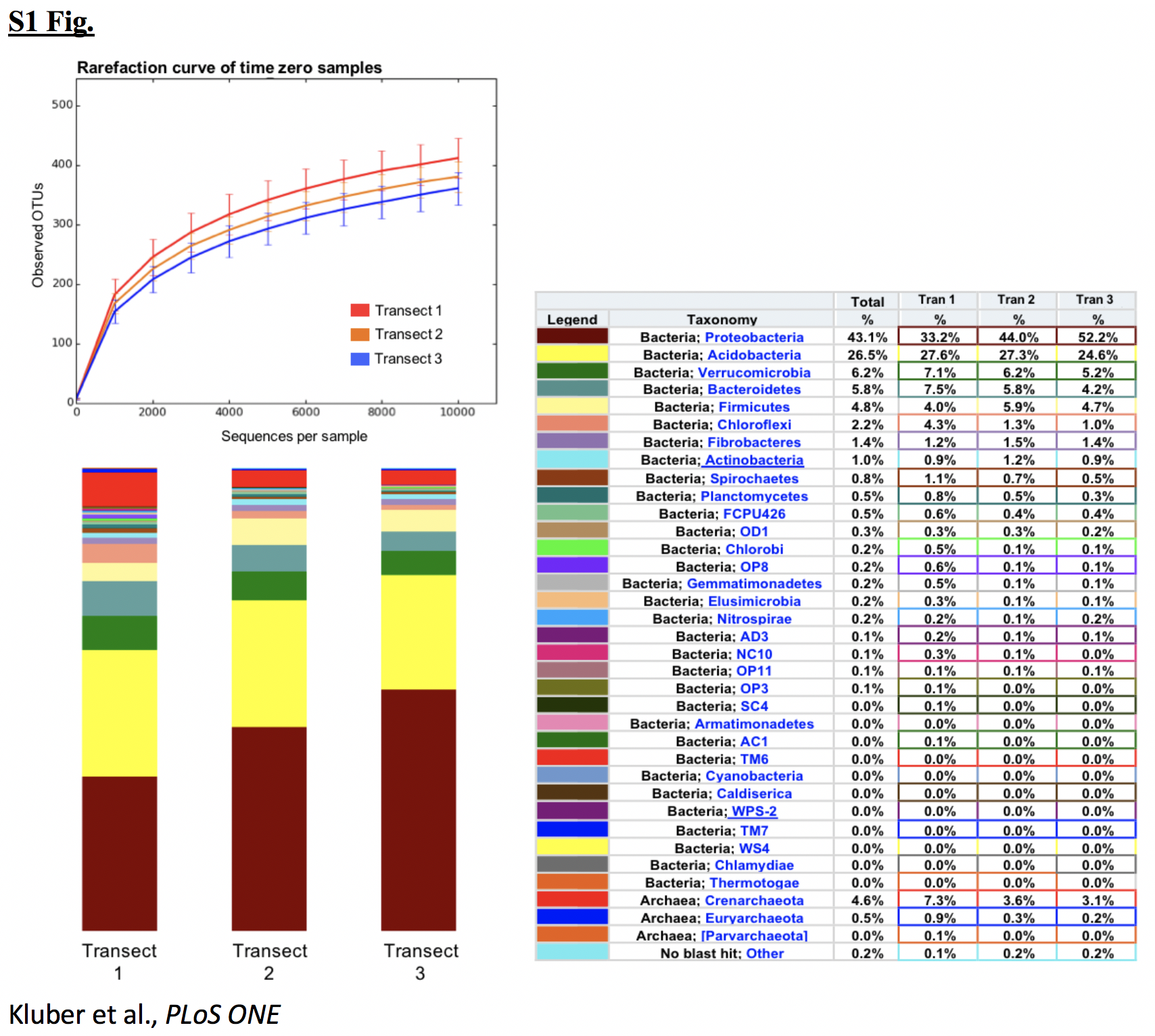

Supplement: S1 Fig — Transect one had greater diversity and a higher relative abundance of Archaeal sequences. (TIFF) [file pone.0223744.s001.tiff]

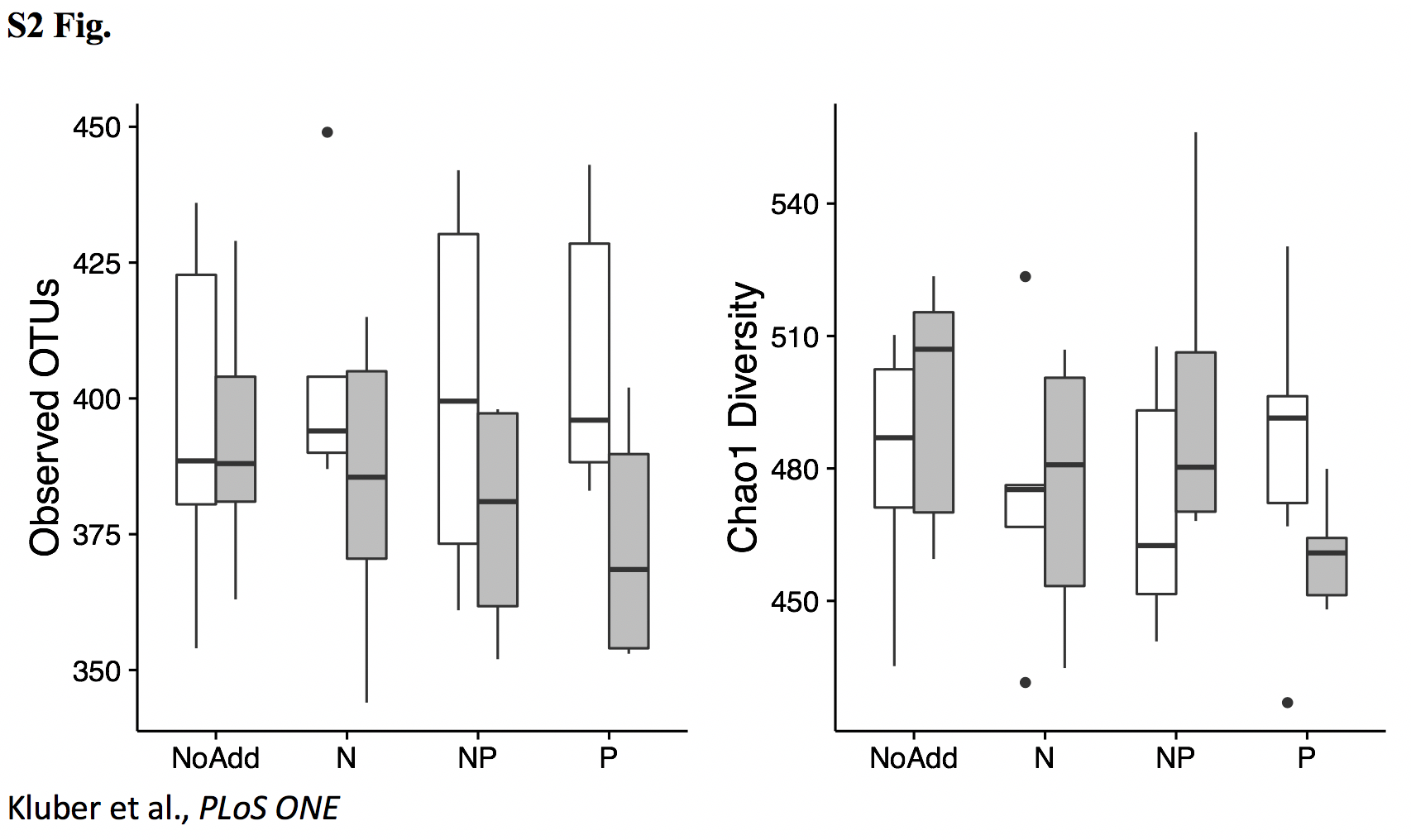

Supplement: S2 Fig — Treatments incubated at 6 and 15°C were not significantly different and are combined for simplicity, thus each bar represents 6 microcosms. White bars represent ambient pH and grey bars elevated pH treatments. (TIFF) [file pone.0223744.s002.tiff]

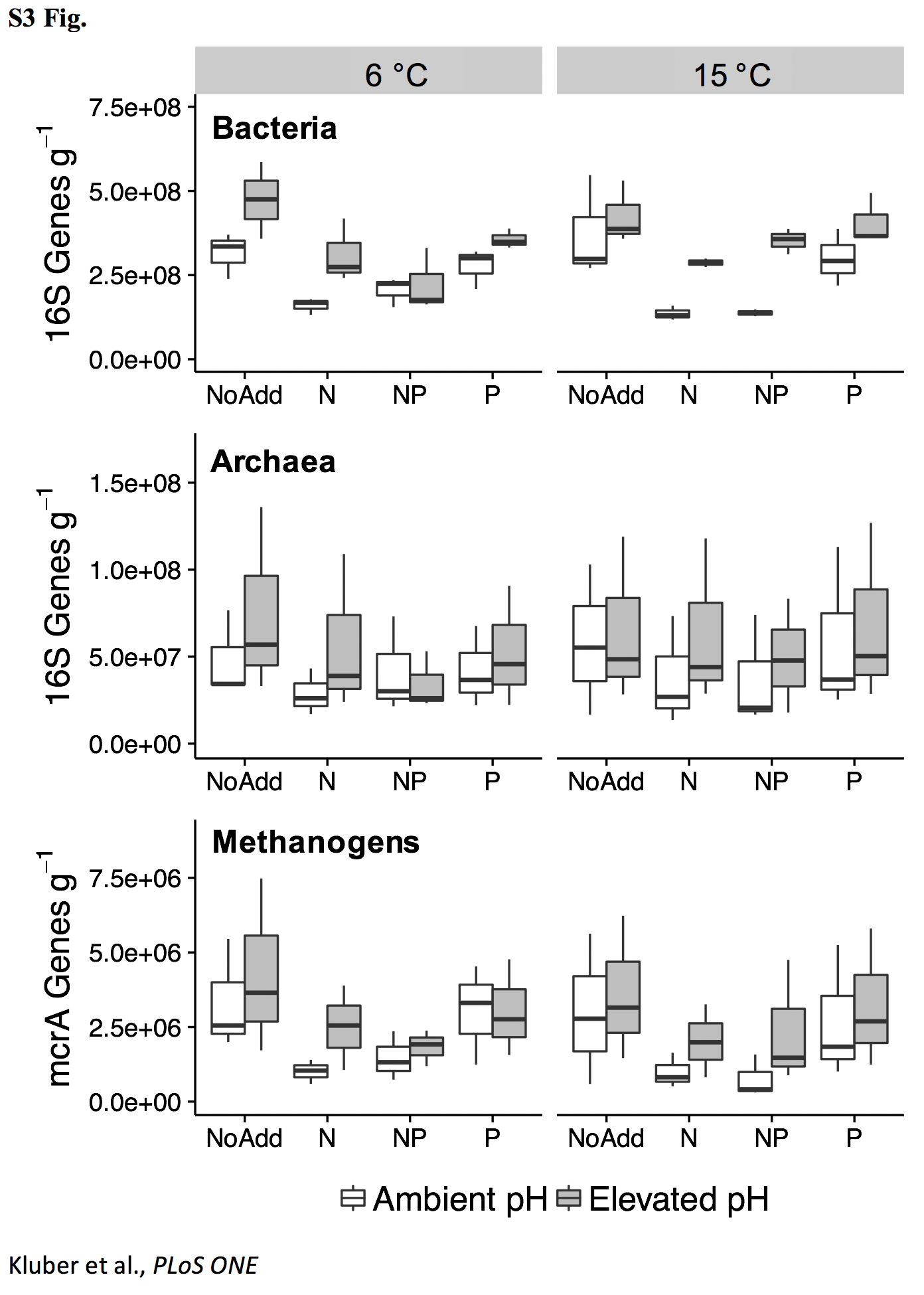

Supplement: S3 Fig — Bacterial and archaeal population size was determined by quantifying 16S rRNA genes and methanogens were assessed by quantifying mcrA gene copies. Abundance is presented as gene copies per g dry peat. (TIFF) [file pone.0223744.s003.tiff]

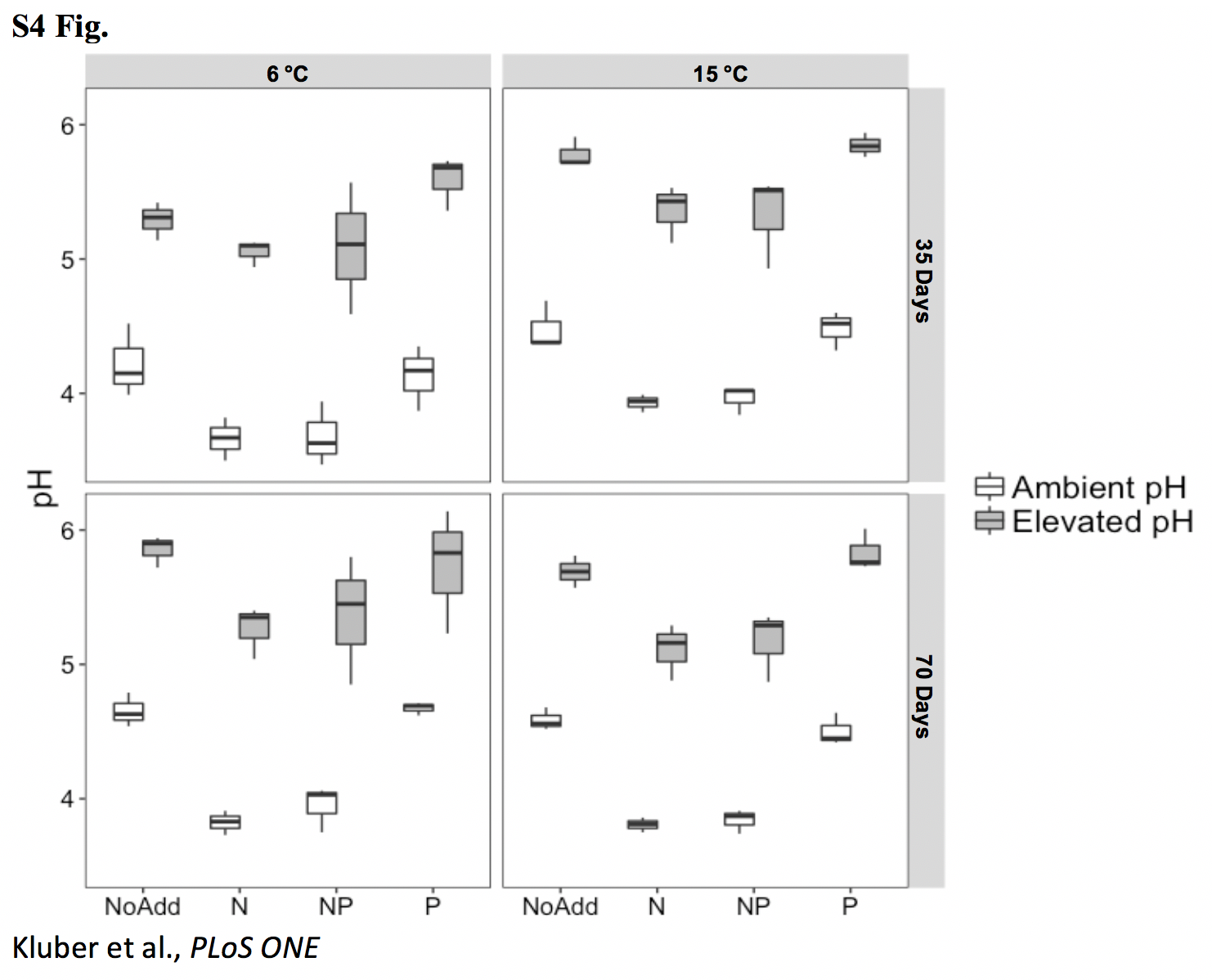

Supplement: S4 Fig — Elevated pH conditions persisted throughout the duration of the experiment. (TIFF) [file pone.0223744.s004.tiff]
