# Supplementary material for: Constraints on microbial communities, decomposition and methane production in deep peat deposits
Source: PLoS One. 2020 Feb 6;15(2):e0223744. doi: 10.1371/journal.pone.0223744 (PMC7004313; doi:10.1371/journal.pone.0223744)
Supplement: S1 Table — (DOCX) [file pone.0223744.s005.docx]

**S1 Table.** **Expanded list of primer mixtures applied for amplicon sequencing.** Adapted for Illumina amplicon sequencing after Lundberg *et al.* 2014.
