# Supplementary material for: Constraints on microbial communities, decomposition and methane production in deep peat deposits
Source: PLoS One. 2020 Feb 6;15(2):e0223744. doi: 10.1371/journal.pone.0223744 (PMC7004313; doi:10.1371/journal.pone.0223744)
Supplement: S2 Table — Bold indicates taxa that had a main effect of pH with no addition interaction. Bacteriodetes was the only phyla with significant main effect temperature differences, and decreased in relative abundance from 6.87% at 6°C to 5.64% (p = 0.04). (DOCX) [file pone.0223744.s006.docx]

**S2 Table.** **Mean relative abundance (and standard deviation) for taxa with significant main-effect p-value for pH.** Bold indicates taxa that had a main effect of pH with no addition interaction. Bacteriodetes was the only phyla with significant main effect temperature differences, and decreased in relative abundance from 6.87% at 6 °C to 5.64% (p= 0.04).

|  | **Ambient pH** | **Elevated pH** | **p-value** |
| --- | --- | --- | --- |
| Acidobacteria | 17.9 (3.54) | 14.1 (6.87) | <0.00 |
| Bacteroidetes | 7.42 (1.83) | 4.96 (2.61) | <0.00 |
| **Chloroflexi** | **2.05 (1.12)** | **1.11 (0.64)** | **<0.00 *** |
| **Fibrobacteres** | **1.60 (0.71)** | **0.95 (0.67)** | **<0.00 *** |
| Proteobacteria | 53.5 (5.89) | 66.5 (11.7) | <0.00 |
| **Alpha** | **8.00 (2.48)** | **5.26 (3.94)** | **<0.00 *** |
| Beta | 34.6 (8.4) | 25.6 (13.6) | <0.00 |
| Gamma | 3.54 (1.86) | 30.5 (27.8) | <0.00 |
| **Verrucomicrobia** | **3.57 (1.22)** | **1.73 (0.82)** | **<0.00 *** |
